# Three-Dimensional Printed Patient-Specific Vestibular Augmentation: A Case Report

**DOI:** 10.3390/jcm13082408

**Published:** 2024-04-20

**Authors:** Linh Johansson, Jose Luis Latorre, Margaux Liversain, Emilie Thorel, Yago Raymond, Maria-Pau Ginebra

**Affiliations:** 1 Biomaterials, Biomechanics and Tissue Engineering Group (BBT), Department of Materials Science and Engineering, Universitat Politècnica de Catalunya BarcelonaTech (UPC), Av. Eduard Maristany, 16, 08019 Barcelona, Spain; linh.johansson@upc.edu; 2 Barcelona Research Centre in Multiscale Science and Engineering, Escola d’Enginyeria de Barcelona Est (EEBE), Universitat Politècnica de Catalunya (UPC), Av. Eduard Maristany, 10-14, 08019 Barcelona, Spain; 3 Biomedical Engineering Research Center (CREB), Universitat Politècnica de Catalunya (UPC), Av. Diagonal, 647, 08028 Barcelona, Spain; 4 Institut de Recerca Sant Joan de Déu (IRSJD), 39-57, 08950 Esplugues del Llobregat, Spain; 5 Mimetis Biomaterials S.L., Carrer de Cartagena, 245, 3E, 08025 Barcelona, Spainsantiago.raymond@envistaco.com (Y.R.); 6 Freelance Implantologist: Oris Dental Center, C. de Joan Güell, 108, 08028 Barcelona, Spain; 7 Institute for Bioengineering of Catalonia (IBEC), BIST, Carrer Baldiri Reixac 10-12, 08028 Barcelona, Spain; 8 CIBER de Bioingeniería, Biomateriales y Nanomedicina, Instituto de Salud Carlos III, 28029 Madrid, Spain

**Keywords:** case report, synthetic, biomaterial, bone grafting, horizontal ridge augmentation, patient-specific, 3D printing

## Abstract

**Background**: The anterior maxilla is challenging regarding aesthetic rehabilitation. Current bone augmentation techniques are complex and 3D-printed bioceramic bone grafts can simplify the intervention. Aim: A four-teeth defect in the anterior maxilla was reconstructed with a 3D-printed synthetic patient-specific bone graft in a staged approach for dental implant delivery. **Methods**: The bone graft was designed using Cone-Beam Computed Tomography (CBCT) images. The bone graft was immobilized with fixation screws. Bone augmentation was measured on CBCT images at 11 days and 8 and 13 months post-surgery. A biopsy sample was retrieved at reentry (10 months post-augmentation) and evaluated by histological and micro-computed tomography assessments. The definitive prosthesis was delivered 5 months post-reentry and the patient attended a visit 1-year post-loading. **Results**: A total bone width of 8 mm was achieved (3.7 mm horizontal bone gain). The reconstructed bone remained stable during the healing period and was sufficient for placing two dental implants (with an insertion torque > 35 N·cm). The fractions of new bone, bone graft, and soft tissue in the biopsy were 40.77%, 41.51%, and 17.72%, respectively. The histological assessment showed no signs of encapsulation, and mature bone was found in close contact with the graft, indicating adequate biocompatibility and suggesting osteoconductive properties of the graft. At 1-year post-loading, the soft tissues were healthy, and the dental implants were stable. **Conclusions**: The anterior maxilla’s horizontal ridge can be reconstructed using a synthetic patient-specific 3D-printed bone graft in a staged approach for implant placement. The dental implants were stable and successful 1-year post-loading.

## 1. Introduction

Tooth extraction or loss, if not treated, triggers a cascade of events, including the loss of buccal tissue and alveolar bone. During the first three months after tooth extraction, the alveolar ridge width is reduced by 50%, corresponding to a loss of 5–7 mm [1]. Due to this atrophy, bone deficiency may occur in the anterior maxilla, causing a lack of support for adequate and stable implant placement, which compromises their function and aesthetics [2,3]. A minimally invasive alternative for placing dental implants in such affected areas is the use of shorter or tilted implants; however, they do not necessarily solve the aesthetic issues [3]. Moreover, the anterior maxilla is the most challenging area regarding aesthetic rehabilitation and most cases require horizontal ridge augmentations to restore the bone plate prior to dental implant placement [4]. 

Today, various techniques are used to achieve ridge augmentation, such as guided bone regeneration (GBR) using bone graft granules as a filler and a membrane for confinement; bone split techniques using chisels and/or osteotomes; bone grafting with standard blocks; and customized bone grafts [3,5,6,7]. Although GBR is a frequently used technique in vestibular augmentations, unconfined granules can migrate, making it difficult to obtain a stable volume during the bone regeneration period. Some methods, such as the sausage technique first introduced by Urban and colleagues [8], aim at achieving stable volumes in large GBR reconstructions. However, the use of this technique demands advanced technical skills from the clinician. Bone split techniques are complex and, in many cases, result in high tissue morbidity. Standard blocks are beneficial for non-confined defects; however, they require onsite milling and shaping, prolonging the surgeries, and often result in a poor fit with the surgical site and high resorption of the graft [3,5,6,7]. One strategy to shorten surgical time and improve the fit of these grafts is based on customized and pre-milled bone blocks using pre-surgical virtual planning [9]. Common materials used for bone grafting with these techniques can be autologous (harvested from a donor site in the same patient), allogenic (collected from a human donor), xenogenic (from animal origin), or alloplastic (synthetically manufactured) [5]. Although autologous bone grafts are considered the gold standard [10,11] due to their osteoconductive, osteoinductive, and osteogenic properties [12], they also have drawbacks, including donor site morbidity, unpredictable resorption, limited available volume and geometrical restrictions, as well as the need for additional surgery sites [12]. Allografts and xenografts can overcome most of these issues; nevertheless, unpredictable resorption remains, with the added risk of rejection, disease transmission, and ethical issues [11,13]. 

Recently, 3D-printed synthetic bone grafts have emerged as an alternative to overcome both technical and material-related issues. This technology can simplify the augmentation procedure, increase the accuracy of the bone graft dimensions, and reduce surgery time. They meet all the advantages of allograft blocks while eliminating the risk of disease transfer, allowing for a precise control of the external shape and volume, which helps predict the final bone gain and the geometrical fit with the contour of the surgical site [7]. Moreover, the internal porosity of the 3D-printed constructs can be controlled and tailored for optimal vascularization and bone colonization, which is an advantage compared to solid pre-shaped blocks [7,11]. Calcium-deficient hydroxyapatite (CDHA) 3D-printed bone grafts stand out among customized synthetic bone grafts. They are obtained by a hydrothermal low-temperature (i.e., 100 °C) dissolution–precipitation process that results in a composition and structure very close to that of the mineral phase of the native bone. They have been proven effective for regenerating large segmental defects in sheep in a preclinical study by Vidal et al. [14]. Furthermore, Raymond et al. [15] and Barba et al. [16] reported bone ingrowth and osteoconductivity when implanted in the femoral condyle in rabbits and in a femoral monocortical defect in beagle dogs, respectively. 

Horizontal ridge augmentations in the anterior maxilla can benefit from this technology, as aesthetic reconstructions are challenging in this area. The current clinical case presents a horizontal ridge augmentation of a four-teeth defect in the anterior maxilla by using a custom-made, 3D-printed bone graft composed of synthetic CDHA, with a staged approach for dental implant placement and a 1-year follow-up (post-loading). 

## 2. Materials and Methods

Prior to implantation and conduction of the analysis for the present case report, the patient was informed and consented to participate. Moreover, this case report was written in accordance with the guidelines of the CARE checklist [17]. Ethics approval is not required for this Case Report since it is not part of a clinical trial and the medical devices used are commercially available with regulatory clearance. 

### 2.1. Case Presentation

A 67-year-old woman with no medical issues or toxic habits suffered from tooth loss. An experienced and specialized maxillofacial surgeon assessed the case and determined the necessity for bone reconstruction of the maxillary vestibular area (positions # 11, 12, 21, and 22) prior to the staged placement of dental implants and final crown prostheses. Following a consultation with the implantologist and a review of the different available options on the market in conjunction with the patient compatibility and the choice of keeping long-term temporary crowns, it was decided that the most appropriate bone grafting solution was a custom-made synthetic scaffold for regenerating the vestibular bone. 

### 2.2. Patient-Specific Bone Graft: Description, Virtual Surgical Planning (VSP), and Design

**Figure 1:**
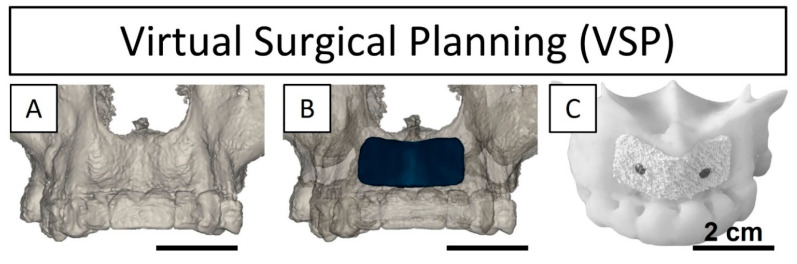
Virtual surgical planning (VSP) before the vestibular bone augmentation surgery. (**A**) Segmented and 3D-reconstructed bone tissue of the patient; (**B**) designed bone graft (dark blue) virtually fitted in the surgical site; (**C**) fixed bone graft with fixation screws in a representative anatomical model of the patient’s bone.

The personalized bone graft was designed and manufactured according to the following process. First, the bone tissue around the defect zone was segmented (Mimics Innovation Suite, Medical, V.25, Materialise, Leuven, Belgium) from the patient’s cone-beam computed tomography (CBCT) images and exported as a stereolithography (STL) file (Figure 1A). Subsequently, a computer-aided design (CAD) of the graft was created based on the segmented bone mesh combined with the surgeon’s design input, aiming at both a geometrical fit with the contour of the bone tissue and the desired bone volume (0.8 cc; 3.7 mm in thickness, 12 mm in height) required for dental implant placement (Figure 1B). The bone graft design STL format was combined with its corresponding printing parameters (defining the scaffold pattern and porosity size and percentage), obtaining a numerical control script (G-code file) (SIMPLIFY3D, V.3.0.2, 2015, Cincinnati, OH, USA). This file was executed by the 3D printer to create a bone graft by direct-ink-writing (DIW), where filaments were extruded and deposited layer-by-layer through a Ø311 µm nozzle (Smooth Flow Tapered Dispensing Tip, Gauge 24, Fisnar, Germantown, WI, USA). Filaments were 3D-printed in an orthogonal pattern (0–90° lay-down) at a deposition velocity of 15 mm·s^−1^ with a strand-to-strand separation of 254 µm and a layer height of 233 µm. The synthetic patient-specific bone graft (MimetikOss 3D, creos syntogain 3D, lot: 3D-20210512-01, Mimetis Biomaterials S.L., Barcelona, Spain) was manufactured with a build material composed of a calcium phosphate extrudable paste. The biomimetic bone graft was composed of 80% calcium-deficient hydroxyapatite (CDHA) and 20% beta-tricalcium phosphate (β-TCP). Prior to the printing of the implantable bone graft, an identical scaffold was printed for design validation, where both geometrical fit and fixation to an anatomical model of the segmented bone (i.e., printed replica of the patient’s bone) were tested (Figure 1C). Finally, the 3D-printed bone graft was packaged in double sterilization pouches and sterilized by moist heat in an autoclave (15 min, 121 °C) before the surgery. 

### 2.3. Surgical Procedures

#### 2.3.1. Vestibular Augmentation

**Figure 2:**
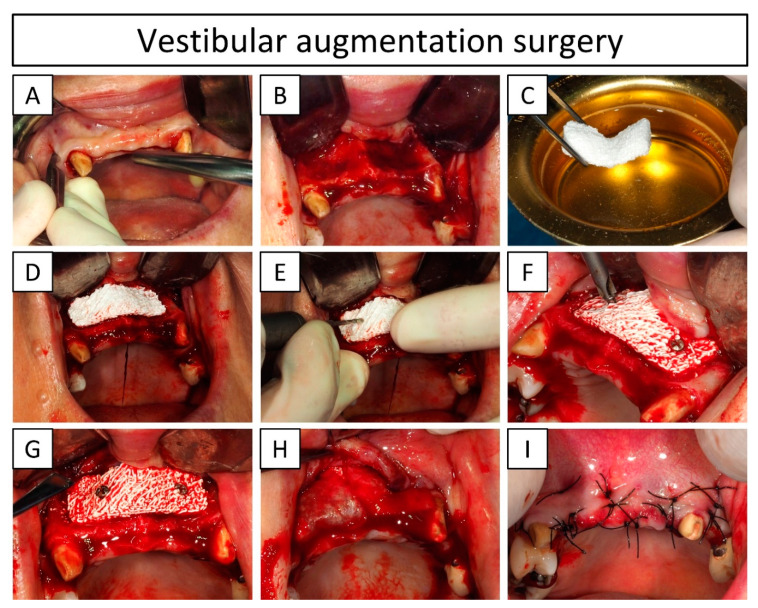
Clinical images demonstrating the vestibular bone augmentation (positions # 11, 12, 21, and 22) with a patient-specific bone graft. (**A**) Opening of the coronally advanced trapezoidal flap in the vestibular area; (**B**) exposed bone perforated with intramedullary holes; (**C**) hydration of the bone graft by immersion in saline solution; (**D**) placement of the bone graft in the surgical site; (**E**) perforation of the bone graft and the pristine bone; (**F**) fixation of the bone graft to the pristine bone with fixation screws; (**G**) fixed and immobilized bone graft; (**H**) covering of the bone graft with resorbable collagen membranes; (**I**) tension-free closure of the soft tissues (prior to closure, an incision in the periosteum was performed to obtain a coronally advanced flap).

Under local anesthesia of the back of the oral vestibule (5.1 mL injectable solution containing 40 mg·mL^−1^ articaine hydrochloride and 0.01 mg.mL^−1^ epinephrine (Ultracaín^®^, Normon S.A., Madrid, Spain), administered via infiltrative local anesthesia), a horizontal gingival incision between the canines and two oblique vertical incisions were performed to create a trapezoidal flap in the vestibule (Figure 2A). 

Once exposed, the bone on the implant site was perforated with a Ø1.2 mm drill bit (Ø1.2 mm × L8 mm drill bit, OsteoMed^®^, Dallas, TX, USA) at 1000–1500 rpm, creating 8 adjacent intramedullary holes activating the bleeding of the bone to promote angiogenesis (Figure 2B). Prior to placement, the custom-made bone graft was hydrated by immersion in saline solution for 5 min (Figure 2C). Thereafter, it was placed on the surgical site, oriented in the only possible way for a perfect fit with the contour of the patient’s bone (Figure 2D). Subsequently, the bone graft together with the pristine bone were perforated to create two adjacent holes 15 mm apart, with a Ø1.2 mm drill (Ø1.2 mm × L8 mm drill, OsteoMed^®^, Dallas, TX, USA) at 1000–1500 rpm (Figure 2E), for graft immobilization with fixation screws. The bone graft fixation holes were enlarged to Ø1.5 mm, using a Ø1.5 mm × L8 mm drill (OsteoMed^®^, Dallas, TX, USA) and rotor at 1000–1500 rpm, to avoid radial tensions on the scaffold upon fixation. Immediately after, two fixation screws (Ø1.5 mm × L8 mm, OsteoMed^®^, Dallas, TX, USA) were alternatively and progressively tightened (Figure 2F) until the bone graft was completely immobilized and fixated to the pristine bone in the defect (Figure 2G). Subsequently, a coronally advanced flap was obtained by performing an incision in the periosteum at the base of the vestibular flap (a key procedure for tension-free closure of the soft tissues). Prior to suturing (4.0, Black pseudo-monofilament: non-absorbable PA, SUPRAMID, Ergon Sutramed srl, Rome, Italy), two resorbable collagen membranes (15 × 20 mm, Straumann^®^ Membrane Flex, Straumann^®^, Basel, Switzerland) were placed over the bone graft (Figure 2H). The soft tissues were closed with three-layer sutures by first performing vertical mattress sutures to release tensions, secondly by adding horizontal mattress stitches to bring the two flaps together, and finally by adding simple stitches to finish the closure (Figure 2I). A provisional prosthesis (D’Acosta Laboratori Dental, Barcelona, Spain) was placed (i.e., a bridge supported by adjacent teeth) immediately after the surgery. Follow-up visits were scheduled at 11 days and 1-, 8-, and 13 months post-surgery. Moreover, the dental implant placement was planned for 10 months post-surgery (expecting complete bone colonization of the implant placement region) and the definitive prosthesis was delivered 5 months after reentry with a planned follow-up at 1 year post-loading. Furthermore, the healing of the soft tissues, adverse events, osseointegration and rehabilitation of the regenerated area, and bone gain were evaluated at each follow-up visit by visual inspection and through CBCT images.

#### 2.3.2. Dental Implant Placement

**Figure 3:**
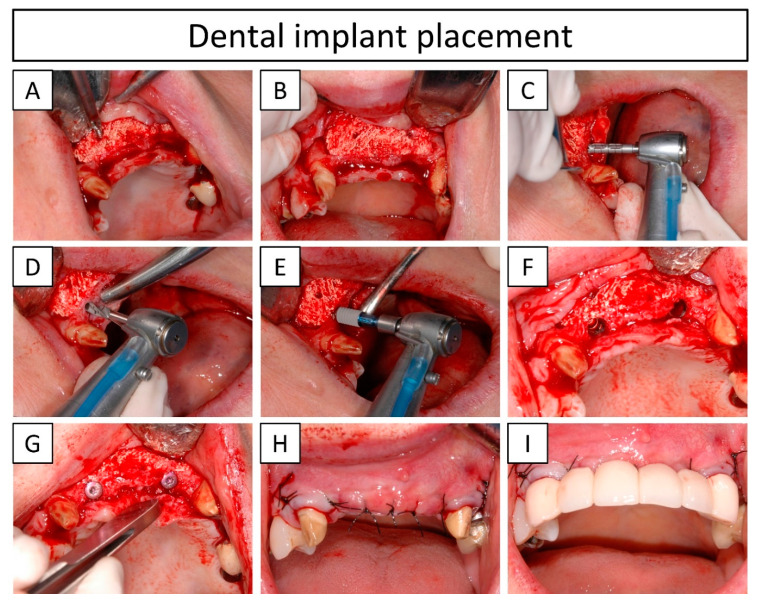
Clinical images showing the reentry with the dental implant placement 10 months post-surgery. (**A**) Removal of the fixation screws placed to immobilize the bone graft during the bone regeneration period; (**B**) regenerated area with removed fixation screws; (**C**) extraction of a biopsy with a trephine burr; (**D**) perforation in the regenerated area for dental implants insertion; (**E**) subcrestal dental implant placement; (**F**) placed implants in positions # 12 and 22, insertion torques are shown in Figure S1, Supplementary information; (**G**) placed closure caps; (**H**) closed flap with loose and simple sutures; (**I**) immediate placement of the provisional prosthesis (i.e., a bridge supported by adjacent teeth).

Two dental implants were placed (positions # 12 and 22) 10 months after the grafting procedure to ensure complete bone colonization of the implant placement region. Under local anesthesia of the back of the oral vestibule (5.1 mL injectable solution containing 40 mg·mL^−1^ articaine hydrochloride and 0.01 mg·mL^−1^ epinephrine (Ultracaín^®^, Normon S.A., Madrid, Spain), administered via infiltrative local anesthesia), the soft tissue flap was reopened following the same procedure as stated in the previous section. Once exposed, the overall aspect of the regenerated zone was evaluated in terms of colonization, osseointegration, and bone quality. First, the two horizontal fixation screws that were placed to immobilize the bone graft during the healing period were removed (Figure 3A,B). Thereafter, trephines (Ø2 mm/Ø3 mm (inner/outer diameter) × L10 mm, © Hager & Meisinger GmbH, Neuss, Germany) were used at 800 rpm to generate the holes for the implant placement and to take advantage to obtain bone biopsies from the interface of the regenerated bone and the host tissue (Figure 3C). The biopsies were preserved in a 10% neutral-buffered formalin solution for later examination. Subsequently, the osteotomy was created in three steps (with accurate hole diameter and length): first with a Ø2.2 mm drill, secondly with a Ø2.8 mm drill, and thirdly with a Ø3.5 mm drill (all from Straumann^®^, Basel, Switzerland) at 800 rpm (Figure 3D), and two subcrestal dental implants (Ø4.1 mm Bone Level Tapered Implant, Regular CrossFit^®^, SLA^®^ 12 mm, Ti, Loxim^®^, Straumann^®^, Basel, Switzerland) were placed (Figure 3E,F). The insertion torque was measured with a torque wrench (046.049, Straumann^®^, Basel, Switzerland), and finally, closure caps (Regular CrossFit^®^, Straumann^®^, Basel, Switzerland) were added to the dental implants (Figure 3G). The coronally advanced flap was closed with loose and simple sutures (4/0, Black non-absorbable PA, Supramid, Suturas Aragó, Laboratorio Aragó, Aragon, Spain) (Figure 3H) and a provisional prosthesis (D’Acosta laboratory dental, Barcelona, Spain) was immediately placed (i.e., a bridge supported by adjacent teeth) (Figure 3I). Definitive prosthesis (D’Acosta laboratory dental, Barcelona, Spain) was delivered 5 months after the reentry. A follow-up visit 1-year post-loading was planned.

### 2.4. CBCT Follow-Up

**Figure 4:**
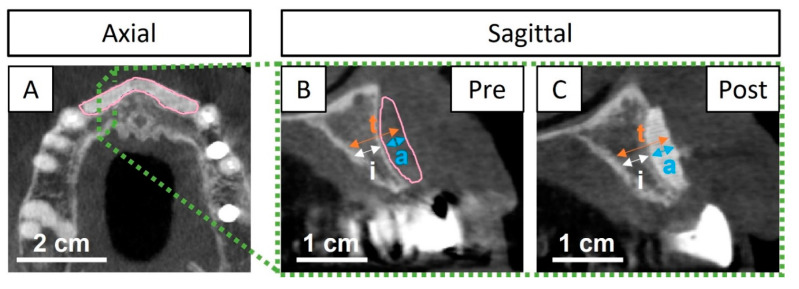
Representative CBCT images to show how the measurements of the bone thickness were performed. The pink line indicates the contour of the pre-designed graft, and the green lines indicate the position and projection where measurements were performed. (**A**) Axial projection with pre- and post-surgery CBCT images superimposed; (**B**,**C**) a sagittal projection indicates how the different measurements were obtained, (**B**) pre-surgery (i.e., initial bone and pink line indicating planned augmentation), (**C**) post-surgery (i.e., initial bone and grafted area): i = initial bone thickness, a = bone thickness augmentation (i.e., gained bone) and t = total bone thickness; t = (i + a).

The bone thickness was measured from post-surgical CBCT images (i.e., after the vestibular bone augmentation procedure) at 11 days and 1-, 8-, and 13 months (i.e., 3 months after dental implant placement). The evaluation was made in axial and sagittal projections using the software Mimics (Mimics Innovation Suite, Medical, V.25, Materialise, Leuven, Belgium). The bone graft adaptation and the gained bone thickness were compared at each time point to the pre-surgical CBCT images. Measurements were taken as described in Figure 4. 

### 2.5. Biopsy Evaluation

The biopsy extracted during the reentry (10 months post-surgery) was fixed in 10% neutral-buffered formalin solution for two weeks and then dehydrated in an increasing series of ethanol solution (70–100%) before micro-computed tomography (μ-CT) and histological (staining) assessments to evaluate the bone ingrowth and activity.

#### 2.5.1. μ-CT Assessment

**Figure 5:**
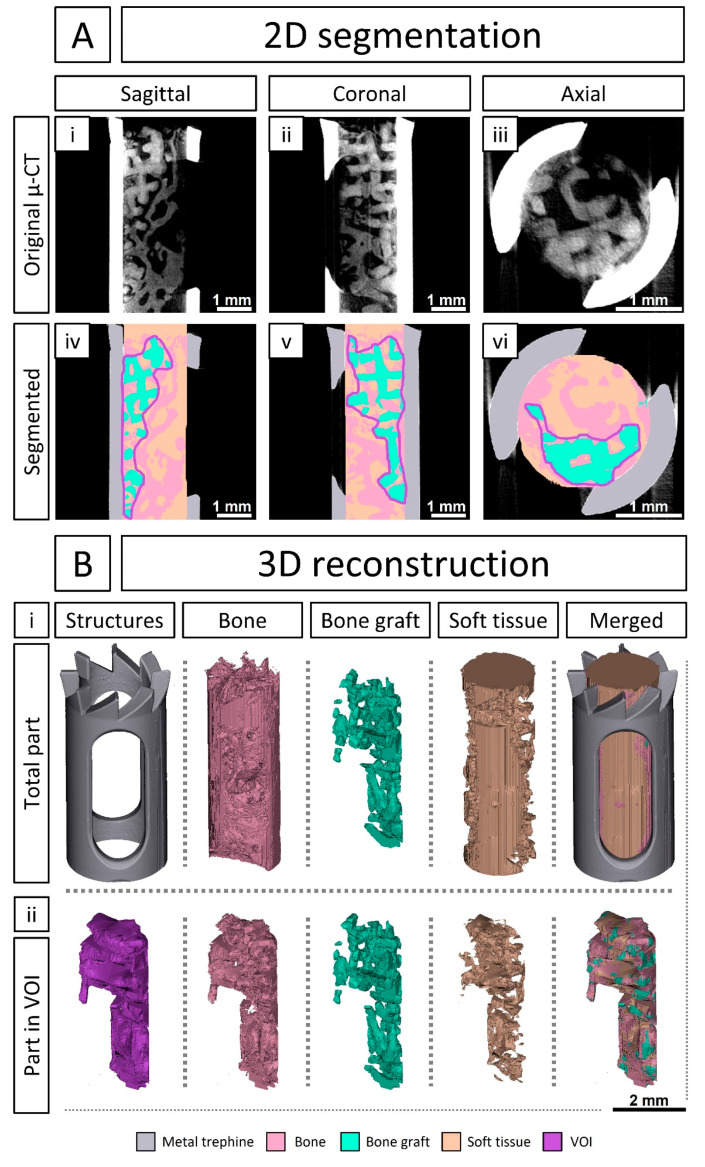
Two-dimensional segmentation and 3D reconstruction of the μ-CT acquisitions obtained from the biopsy extracted at the reentry (10 months post-surgery). (**A**) Two-dimensional segmentation in sagittal, coronal, and axial projections: (**Ai**–**Aiii**) slice images of the stack before segmentation, (**Aiv**–**Avi**) segmented images with the four model sub-populations distinguished by different masks: in grey: metal trephine, in pink: bone, in green: bone graft, in orange: soft tissue, and in purple line: the contour and limits of the volume of interest (VOI); (**B**) 3D reconstruction of the segmented image stack (in grey: metal trephine, in pink: bone, in green: bone graft, in orange: soft tissue, and in purple: VOI): (**Bi**) complete volume of the segmented parts, (**Bii**) volume of each region inside the VOI.

The biopsy was analyzed by micro-computed tomography (μ-CT) (Skyscan 1272, Bruker, Billerica, MA, USA) with an X-ray beam with a 0.11 mm thick copper filter. The X-ray tube current and acceleration voltage were set to 100 μA and 100 kV, respectively. A 360° rotation was used in combination with an exposure time and step resolution of 2625 ms and 0.2° per step, respectively. The frame averaging applied was 3 and the acquisition was performed with an isotropic voxel resolution of 6.5 μm. The data were treated with beam hardening correction and object shifting correction before further reconstruction into tomographic image stacks in Digital Imaging and Communications in Medicine (DICOM) format (NRecon software, V2.0, Bruker, Billerica, MA, USA). The image stacks were imported and analyzed in the software Mimics (Mimics Innovation Suite, Medical, V.25, Materialise, Leuven, Belgium), where the different regions (i.e., metallic trephine, new and pristine bone, bone graft, and soft tissues) of the biopsy were segmented and represented by separate colored masks (Figure 5A). Based on the previous 2D segmentation, a 3D model consisting of the different parts was reconstructed and visualized digitally (Figure 5B). Finally, the percentages of bone, bone graft, and soft tissue in the biopsy were calculated in the defined volume of interest (VOI), contouring the outline of the bone graft. Thus, only the regenerated area was evaluated, in order not to overestimate the portion of bone in the sample. 

#### 2.5.2. μ-CT Assessment

After the μ-CT acquisition, the dehydrated trephine biopsy was infiltrated with different graded ethanol–glycol methacrylate (Technovit 7200 VLC, Heraeus Kulzer, Wehrheim, Hanau, Germany) mixtures with increasing concentrations of glycol methacrylate in each series. Samples were held for 24 h under agitation in each solution. Subsequently, the sample was polymerized at 37 °C for 24 h. Once completely polymerized, thin slides were obtained through the method described by Donath [18] and ground using a grinding machine (Exakt Aparatebau, Hamburg, Germany) and P1200 to P4000 grid silicon carbide papers (Struers, Copenhagen, Denmark) until 40 μm thickness was achieved. Thereafter, the slides were treated with the Levai–Laczkó staining method for bone mineralization detection [19]. Images were acquired using an optical microscope (BX51, Olympus, Tokyo, Japan) connected to a digital color camera (DP71, Olympus, Tokyo, Japan) with a motorized plate (Märzhäuser, Steindorf, Germany). Images were acquired at magnifications of 40×, 100×, and 200×. 

## 3. Results

### 3.1. Case Follow-Up

#### 3.1.1. General Aspects and Radiological Assessment

**Figure 6:**
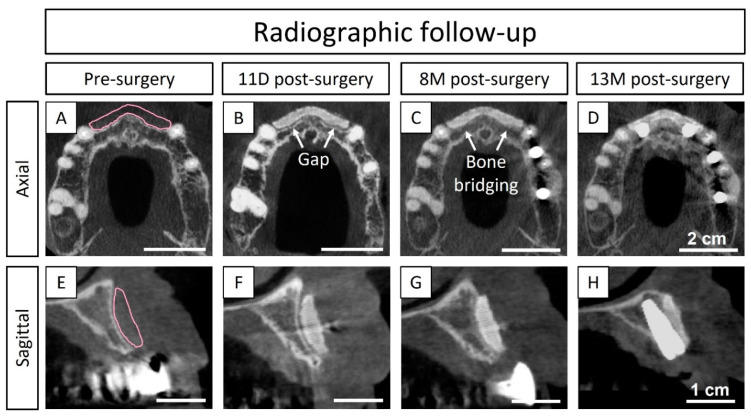
CBCT images acquired pre-surgery, 11 days, 8 months, and 13 months post-surgery (3 months after reentry and dental implant placement). (**A**–**D**) Axial projection; (**E**–**H**) sagittal projection, used for bone thickness measurements: (**A**,**E**) pink lines indicating the pre-designed bone graft and planned bone augmentation, (**B**,**F**) revealing a gap between the bone graft and the pristine bone, (**C**,**G**) demonstrating new bone ingrowth and bridging of the previous gap, (**D**,**H**) dental implant position after successful bone augmentation and implant placement.

A 3D-printed patient-specific synthetic bone graft was implanted for a vestibular augmentation to increase bone volume (the planned augmentation is shown in Figure 6A,E) and restore the bone function to further be able to place stable dental implants. The 3D-printed bone graft resulted in a perfect fit with the contour of the host bone and showed an instantaneous absorption of the patients’ blood when placed in the defect. Moreover, the pre-operatory planning allowed for the shortening of the implantation and fixation time to only 10 min. There were no adverse events during the surgery and the coronally advanced flap allowed for a complete tension-free closure of the soft tissues. 

At 11 days post-surgery, the wound was well closed, and CBCT images showed a good adaptation of the implanted graft (Figure 6B,F). However, a slight gap in certain areas of the interface between the graft and the bone was observed. At 19 days post-surgery, some of the stitches were removed and the rest of the sutures were removed 1 month post-surgery. The wound stayed closed without any exposure of the bone graft. At 8 months post-surgery, CBCT showed closure of the gap by newly formed bone (Figure 6C,G), thus revealing bone bridging and osseointegration of the bone graft with the host tissue. The two dental implants were placed (positions # 12 and 22) 10 months after the bone augmentation procedure. At the reentry, the bone quality was evaluated as a type-III according to the Lekholm–Zarb classification (i.e., a thin layer of cortical bone surrounding a core of trabecular bone of good strength) [20] and a well-colonized and osseointegrated scaffold was observed (later confirmed by μ-CT and histological assessments). Moreover, the bone sounded and had a hard aspect when scrapping the regenerated area with a spatula. The bone graft was still visible at reentry, confirming the slow degradation rate of the CDHA. Nevertheless, the regenerated area showed a uniform and hard bone block, and the bone graft was impossible to separate from the pristine bone. The dental implants were placed at the interface of the regenerated area and the pristine bone without any complications and resulted in an insertion torque greater than 35 N·cm for both positions (# 12 and 22) (Figure S1, Supplementary information), which was the measurement limit of the instrument, confirming the previously observed hard aspect of the regenerated bone tissue and an early stability for the placed implants. CBCT at 3 months after reentry and dental implant placement (i.e., 13 months post bone augmentation procedure) showed that both dental implants were stable (Figure 6D,H). 

#### 3.1.2. Radiological Quantification

The bone thickness measurements from the CBCT images in Figure 6 are displayed in Table 1. The initial bone thickness (i.e., before surgery) was 4.3 mm and the target bone thickness augmentation was pre-planned to 3.7 mm, in order to achieve a total bone thickness of 8.0 mm at the reentry and implant placement. At 11 days post-bone augmentation, the achieved thickness was 3.6 mm, 0.1 mm less than planned, which could be due to the small gap observed in the CBCT images (Figure 6B,F). Nevertheless, at 8 months, the target thickness was achieved, due to the bridging of the newly formed bone, and maintained at 13 months (3 months post-reentry and implant placement), resulting in a total bone thickness of 8.0 mm. Restoration of the bone thickness at reentry was successful and adequate for dental implant placement and in accordance with the initial treatment plan (i.e., without changing the dental implant diameter or type, nor requiring complementary bone augmentation). 

### 3.2. μ-CT Assessment

A biopsy was extracted with a trephine during the reentry at 10 months post-surgery. After adequate preparation of the sample, a detailed assessment of the bone ingrowth and soft tissue fraction in the regenerated area was made from the μ-CT analysis. The percentage of new bone, bone graft, and soft tissue in the regenerated area was determined in the reconstructed and segmented image stack of the biopsy (Figure 5) and is displayed in Table 2. A higher presence of mature bone compared to soft tissue was observed in the available volume (i.e., 69.7 vs. 30.3% in the macropores, respectively) and the amount of newly formed bone was similar to the bone graft volume in the regenerated area. 

### 3.3. Histological Assessment

**Figure 7:**
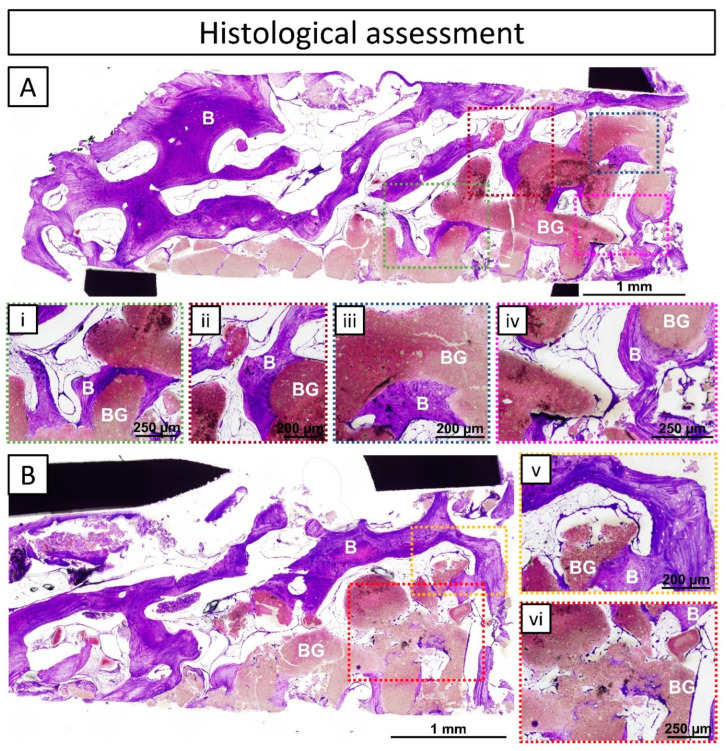
Histological assessment of a biopsy collected 10 months post-surgery (during reentry) from the interface of the pristine bone and the regenerated area. In black: metal trephine, in purple: mature bone = B, in pink: bone graft = BG, and in white: bone marrow. (**A**,**B**) Overviews of the complete trephine sample; (**i**–**vi**) insets showing specific areas of the histological images in a greater magnitude and more detail: (**i**–**v**) new bone growing on the surface of the bone graft and following its contour together with bone marrow regions; (**vi**) signs of local graft and bone remodeling.

The histological assessment of the extracted trephine biopsy showed two different regions in the sample: (I) the patient’s original pristine bone, represented by lamellar bone (well-organized bands of bone growth) alternated with areas of bone marrow; and (II) the regenerated area composed of regenerated tissue (lamellar bone and bone marrow) and bone graft (Figure 7A,B). A layer of newly formed mature lamellar bone grew following the contour of the printed filaments of the graft together with bone marrow regions, which is a clear sign of osteoconduction (Figure 7(i–v)). Additionally, neither changes in tissue morphology, nor inflammatory cells, traumatic necrosis, foreign debris, fatty infiltrate, granuloma, or encapsulation were observed, which is a sign of biocompatibility of the bone grafting material (Figure 7). Moreover, the bone graft appeared stable with no signs of bulk resorption or bone loss. Nonetheless, in certain areas, signs of local graft and bone remodeling were observed (Figure 7(vi)). 

### 3.4. Definitive Prosthesis Delivery and 1-Year Follow-Up

**Figure 8:**
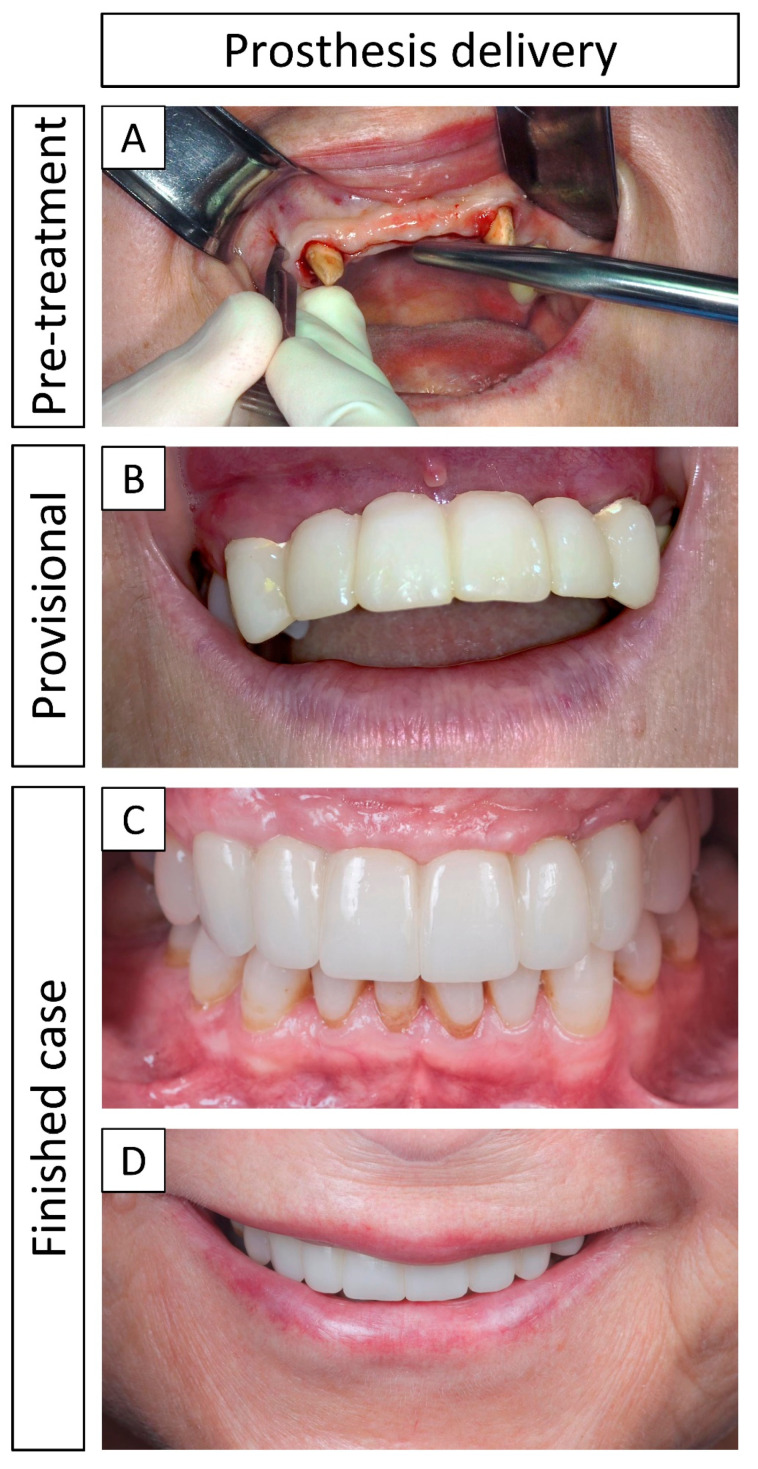
Clinical images of the prosthesis delivery. (**A**) Initial state, before starting the rehabilitation treatment; (**B**) provisional prosthesis (i.e., a bridge supported by adjacent teeth) placed immediately after the bone augmentation surgery; (**C**) definitive prosthesis delivered 5 months after reentry and dental implant placement; (**D**) 1-year follow-up post-loading.

Figure 8 shows pre- and post-treatment images. A provisional prosthesis (i.e., a bridge supported by adjacent teeth) was placed immediately after the bone augmentation procedure (Figure 8B). The definitive prosthesis was delivered 5 months after reentry and dental implant placement (Figure 8C) and at the 1-year follow-up (i.e., 12 months post-loading), the soft tissues were healthy and both implants were stable and well supported by the restored bone (Figure 8D). 

Overall, no inflammatory responses or infections were observed during or after the surgical procedures (including the bone grafting and the dental implant placement), and the final result was positive both in terms of re-established bone function and from an aesthetic perspective. According to the patient, the complete process to finalize the treatment was extensive, but nonetheless worth the wait. The patient was especially satisfied with the final aesthetic result and relieved that the bone and dental functions were completely restored, in the sense that she could finally use her anterior teeth to their full potential. 

## 4. Discussion

In the current clinical case, a patient-specific synthetic 3D-printed bone graft was used for horizontal ridge augmentation of the anterior maxilla, involving a four-teeth defect (positions # 11, 12, 21, and 22) and a staged approach for dental implant placement. Complete osseointegration was observed after 8 months post-surgery with an achieved bone gain of 3.7 mm, as evidenced by CBCT images. At reentry (10 months after the bone augmentation procedure), two stable implants were delivered (both with an insertion torque of above 35 N·cm) and histological and µ-CT assessments on a biopsy retrieved at this time point revealed mature bone ingrowth in the regenerated area. 

The bone graft fitted perfectly in the bone defect, following the contour of the bone in the surgical site, as a result of an accurate virtual surgical planning (VSP) and pre-surgical bone graft design (Figure 1). Moreover, the grafting procedure (including placement and fixation) only took 10 min, compared to 2 h for standard blocks [7,21,22] due to the need for trimming the block in the operating room (OR), or 1 h required with the sausage technique, which additionally demands special technical skills. Nonetheless, it is worth noting that the time required for graft implantation with the different bone augmentation techniques depends on the indication, patient anatomy, and previous experience of the clinician. Vente et al. [9] demonstrated in a case series from 2017 that employing VSP as a pre-operatory strategy improved both the geometrical fit of allogenic bone blocks and reduced surgery time in six consecutive patients (11 block grafts, 12 placed dental implants), due to the pre-drilling, shaping, and fitting in anatomical 3D-models before surgery. Moreover, ensuring a perfect fit between the bone graft and the surgical site prevented the need for adding granules or autologous chips to fill unwanted voids in the reconstruction area, which is a common procedure when using non-personalized grafts [3]. 

The sutures were entirely removed 1 month after the surgery, when the soft tissues were healed, and the wound completely closed. This evidenced correct soft tissue management (i.e., coronally advanced flap by an incision in the periosteum) with tension-free closure of the wound, which is important since the most common complication in ridge augmentation procedures is the exposure of the graft due to soft tissue dehiscence [4,23], especially when using bone blocks, which later can lead to infection. Urban et al. [24] reported in a systematic review and meta-analysis that 23.9% of bone blocks were exposed post-implantation, leading to the conclusion that adequate soft tissue handling is crucial for successful rehabilitation. Another important parameter is the fixation and immobilization of the bone graft, as a second common complication is the loosening of fixation screws and micromovements, which can lead to insufficient osseointegration and graft failure during dental implant placement [25]. 

Dental implants were placed with an insertion torque greater than 35 N·cm (Figure S1, Supplementary Information) in a staged approach 10 months post-surgery (with a provisional prosthesis) and the definitive prosthesis was placed 5 months later. Taking into account the quality of the newly formed bone in the regenerated area observed at 10 months post-surgery (Figure 6 and Figure 7), in terms of maturity and arrangement, as well as the bone bridging between the recipient site and the bone graft at 8 months post-surgery (Figure 6C,G), the reentry at 10 months post-surgery may be judged as a conservative decision. Thus, in future cases, an earlier reentry can be considered. Nonetheless, reentry is usually performed between 4–12 months ± 1 month post-surgery with bone blocks and in horizontal ridge augmentations [4,26]. Shorter times are more common with autologous grafting, while mineral blocks are usually reentered at later time points. Moreover, Vente et al. [9] reported reentry at 6 months post-surgery in horizontal ridge augmentations with allograft blocks. 

Kuchler and von Arx [4] reported in a systematic review that the mean horizontal bone gain achieved in the anterior maxilla with blocks of different origins ranged between 3.4–5.0 mm upon reentry. In the current case, a bone gain of 3.7 mm was achieved with a 3D-printed bone graft, giving a total bone thickness of 8.0 mm as initially planned (Table 1). This indicates that a sufficient volume of bone was gained for the placement of dental implants, eliminating the need for regrafting. Regrafting at reentry is often a necessity due to bone loss during the healing period [3]. Sanz-Sánchez et al. [23] stated in a systematic review (including 40 studies, with 15 studies including regrafting in their outcomes) that 7 out of 15 studies reported the need for regrafting (ranging between 0–23% of the cases) upon reentry in horizontal ridge augmentations with blocks (autologous, allogenic, and xenogenic). However, further long-term results from comparative studies with 3D-printed bone grafts are crucial before establishing a tendency regarding their expected outcomes [3]. 

The potential for bone reconstruction is also influenced by the specific anatomy of the indication and the anticipated bone volume augmentation necessary to restore proper function for the placement of dental implants. In the present case, a four-teeth defect was reconstructed with a 3D-printed bone graft, which was considered to be more promising for this indication than GBR, commonly limited to defects involving one to three teeth [3]. 

µ-CT of the biopsy extracted at 10 months post-surgery revealed that the regenerated area was composed of equal amounts of newly formed mature bone and bone graft material, with 17.72% soft tissue (Table 2). Moreover, the distribution of the newly formed bone revealed bone ingrowth in the macropores between the 3D-printed strands, penetrating throughout the entire bone graft and not only in the periphery, which may be an issue with non-autologous blocks. Sohn et al. [11] reported that bone fusion mainly occurs on the junction, while mostly dead trabeculae are found at the innermost part when using non-autologous bone blocks. Furthermore, the histological assessment of the biopsy (10 months post-surgery) (Figure 7) demonstrated the adequate biocompatibility and osteoconductivity of the bone graft with its close contact with newly formed lamellar bone and its participation in the bone remodeling process. Good osseointegration and osteoconductivity were previously reported for similar bone grafts in preclinical studies in large segmental defects in sheep by Vidal et al. [27], femoral monocortical defects in beagle dogs by Barba et al. [16], and in the tibia of rabbits by Raymond et al. [15]. 

The definitive prosthesis was delivered 5 months after reentry and placement of the dental implants. At the 1-year follow-up (12 months post-loading), the soft tissues appeared healthy and both dental implants were stable and considered successful. Kuchler and von Arx [4] reported prosthesis delivery within 4–6 months after reentry in their systematic review, in which timeframe the final prosthesis was delivered in our case, and Vente et al. [9] delivered the final prosthesis 4 months after reentry in their case series. 

This clinical case provides clinical evidence for the suitability of custom-made synthetic 3D-printed bone grafts for horizontal reconstructions in the ridge involving a four-teeth defect, leading to successful rehabilitation of the anterior maxilla and allowing for stable and successful placement of dental implants, as observed in the 1-year follow-up (post-loading). 

This case report highlights the benefits of using 3D-printed patient-specific bioceramic bone grafts. However, some limitations and challenges still remain when working with such medical devices. First, there are process-related limitations, as on-demand medical devices require individual manufacturing procedures and do not allow for serial production, which increases the overall lead time. Moreover, it is necessary to conduct a VSP to ensure a good geometric fit, which has the direct benefit of reducing surgical time. But it also represents a paradigm shift in surgical workflow. This implies more involvement of the clinician in the pre-operative stages and requires an additional skilled CAD designer, which generally increases the cost of the bone graft. Another limitation, related to the material composition, is the inherent brittleness of bioceramics, which restricts these bone grafts to non-loadbearing applications. One of the main challenges encountered when bringing 3D-printed, patient-specific bone grafts to the market is the regulatory requirements. With the entry into force of the European Medical Device Regulation (MDR, Regulation (EU) 2017/745), manufacturers are required to provide stronger clinical evidence for each indication and intended use, which is challenging with such versatile devices [7]. 

Future opportunities for advances in bone regeneration with this technology include overcoming the brittleness of bioceramic materials, where synthetic composite materials are one possible research line [28]. Moreover, there is a need to enhance the osteogenic properties of synthetic bone grafts (e.g., by incorporating biologically active agents) to meet equal biological performance as autologous bone. Another opportunity for improvement is the printing resolution, in order to achieve precise control of the printed strands in such a way that hierarchical structures with load-optimized architectures, similar to the one in the natural bone, can be achieved. Finally, there is a need for the development of effective soft tissue expanders to avoid dehiscence and exposure of the bone graft, which is a recurrent issue in dental indications, during the healing period.

## 5. Conclusions

This case report describes a successful horizontal bone augmentation in the anterior maxilla using a synthetic 3D-printed, patient-specific bone graft (without the addition of osteogenic enhancements) with a staged approach for dental implant placement. The dental implants showed stability 1-year post-loading. The bone graft was fully osseointegrated and vascularized at reentry as confirmed by μ-CT and histological assessments on a biopsy from the regenerated area extracted at 10 months post-surgery and through follow-up CBCT. These findings demonstrate the suitability of such grafts for bone reconstruction, rehabilitation of the bone function, and aesthetic restoration of the maxilla. 

## Figures and Tables

**Table 1:** Measurements of the bone thickness, revealing the initial bone thickness and bone gain after the vestibular augmentation procedure. Measurements performed in sagittal projection of CBCT images (Figure 6) from pre-surgery, 11 days, 8 months, and 13 months post-surgery (3 months after reentry and dental implant placement).

[mm]	Initial Bone Thickness (i)	Bone Augmentation (a)	Total Bone Thickness (t)
Pre-surgery	4.3	3.7 (planned)	4.3
11 D post-surgery	-	3.6	7.9
8 M post-surgery	-	3.7	8.0
13 M post-surgery	-	3.7	8.0

**Table 2:** Volume and fraction of new bone, bone graft, and soft tissue found in the regenerated area (i.e., inside the volume of interest (VOI)) calculated from a biopsy extracted 10 months post-surgery and analyzed by μ-CT.

	New Bone	Bone Graft	Soft Tissue	VOI
Volume [mm^3^]	1.912	1.947	0.831	4.689
Fraction [%]	40.77	41.51	17.72	-

## Data Availability

The original contributions presented in the study are included in the article. Further inquiries can be directed to the corresponding author.

## Supplementary Materials

The following supporting information can be downloaded at: https://www.mdpi.com/article/10.3390/jcm13082408/s1, Figure S1: Showing the insertion torque of the two dental implants during placement. (**A**) Position # 12; (**B**) Position # 22. Both dental implants had an insertion torque over 35 N.cm.

## Abbreviations

α-TCP	Alpha-tricalcium phosphate
β-TCP	Beta-tricalcium phosphate
µ-CT	Micro-computed tomography
3D-printed	Three-dimensional-printed
CaP	Calcium phosphate
CBCT	Cone-beam computed tomography
CDHA	Calcium-deficient hydroxyapatite
DICOM	Digital imaging and communications in medicine
DIW	Direct ink writing
GBR	Guided bone regeneration
HA	Hydroxyapatite
MDR	Medical device regulation
OR	Operating room
STL	Stereolithography
VOI	Volume of interest
VSP	Virtual surgical planning
