# Supplementary material for: Three-Dimensional Printed Patient-Specific Vestibular Augmentation: A Case Report
Source: J Clin Med. 2024 Apr 20;13(8):2408. doi: 10.3390/jcm13082408 (PMC11051386; doi:10.3390/jcm13082408)
Supplement: Supplementary file 1 [file jcm-13-02408-s001.zip › jcm-2944792-supplementary.pdf]

Journal of
Clinical Medicine

SUPPLEMENTARY INFORMATION

Case Report

Three-Dimensional Printed Patient-Specific Vestibular Augmentation: A Case Report

Linh Johansson [1,2,3,4,5], Jose Luis Latorre [6], Margaux Liversain [5], Emilie Thorel [5], Yago Raymond [5] and Maria-Pau Ginebra [1,2,3,7,8,*]

Citation: Johansson, L.; Latorre, J.L.; Liversain, M.; Thorel, E.; Raymond, Y.; Ginebra, M.-P. Three-Dimensional Printed Patient-Specific Vestibular Augmentation: A Case Report. *J. Clin. Med.* **2024**, *13*, 2408. https://doi.org/10.3390/jcm13082408

Academic Editors: Giovanni Salzano and Luis M. Junquera

Received: 17 March 2024
Revised: 10 April 2024
Accepted: 16 April 2024
Published: 20 April 2024

Copyright: © 2024 by the authors. Licensee MDPI, Basel, Switzerland. This article is an open access article distributed under the terms and conditions of the Creative Commons Attribution (CC BY) license (https://creativecommons.org/licenses/by/4.0/).

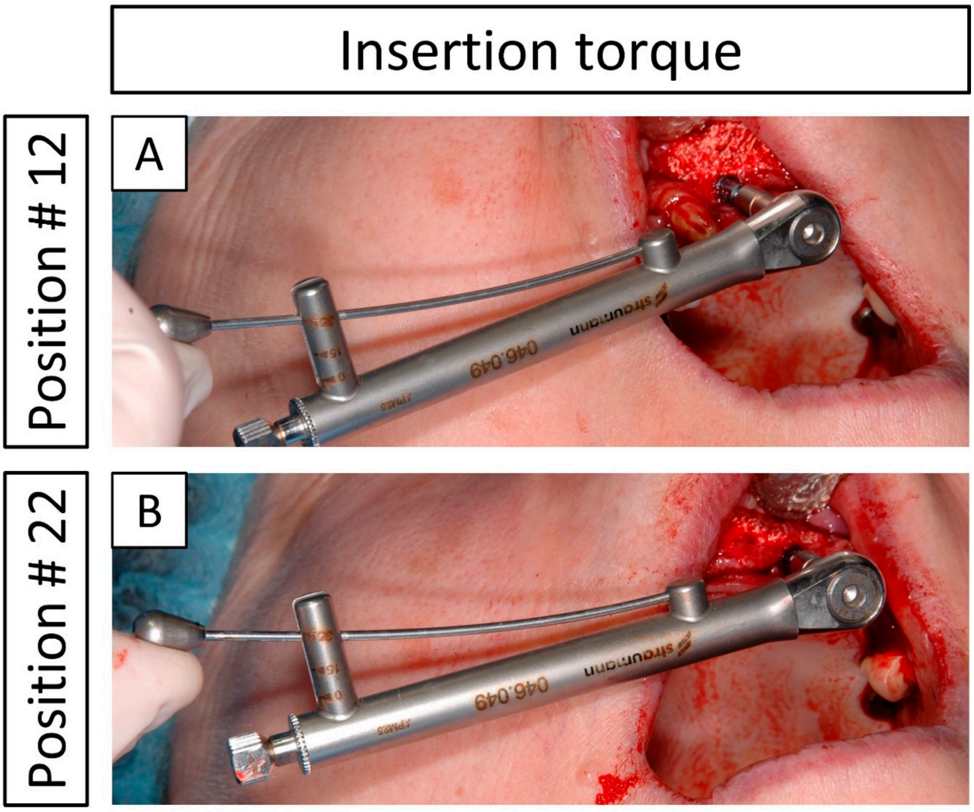

Figure S1. Showing the insertion torque of the two dental implants during placement. (**A**) Position # 12; (**B**) Position # 22. Both dental implants had an insertion torque over 35 N.cm.
